# Persimmon By-Products: Bioactive Phytochemicals, Sustainable Valorization and Potential Food and Health Applications

**DOI:** 10.3390/nu18193194

**Published:** 2026-09-27

**Authors:** Huiwen Wang, Weishi Hou, Haseeb Anwar, Muhammad Sajid Arshad, Malik Adil Nawaz, Hafiz Ansar Rasul Suleria

**Affiliations:** 1 Faculty of Science, School of Agriculture, Food and Ecosystem Science, The University of Melbourne, Parkville, VIC 3010, Australiam.nawaz@uq.edu.au (M.A.N.); 2 School of Agriculture, Environmental, and Veterinary Sciences, Charles Sturt University (CSU), Wagga Wagga, NSW 2650, Australia; 3 School of Agriculture and Food Sustainability, The University of Queensland, St Lucia, QLD 4072, Australia

**Keywords:** persimmon by-products, bioactive compounds, food innovation, food valorization, circular bioeconomy, health-promoting ingredients

## Abstract

Persimmon (*Diospyros kaki* L.) has gained increasing attention as a functional fruit due to its rich bioactive phytochemicals; however, a substantial proportion of its biomass, including peels, seeds, leaves, and calyx, remains underutilized. This review comprehensively examines the phytochemical composition, biological activities, and potential applications of persimmon by-products, with particular emphasis on their role in sustainable food systems and value-added product development. A comprehensive review of the literature was conducted across major databases, including Scopus, Web of Science, PubMed, and ScienceDirect, covering studies published between 2000 and 2025. The findings indicate that persimmon by-products are rich sources of phenolic acids (e.g., gallic, caffeic, and ferulic acids), flavonoids (e.g., quercetin and catechin derivatives), carotenoids, tannins, dietary fibre, and bioactive lipids. These compounds exhibit diverse biological activities, including antioxidant, anti-inflammatory, antidiabetic, hypolipidemic, and antimicrobial effects, with the available evidence being primarily derived from in vitro and in vivo studies. Additionally, recent research highlights their potential applications in food products, food supplements, nutraceutical research, and other health-related products. Despite these promising findings, limitations related to variability in extraction methods, lack of standardized assays, and limited clinical validation remain significant challenges. Overall, this review provides a comprehensive synthesis of current knowledge and identifies key research gaps to support the future evaluation and responsible utilization of persimmon by-products in food and health-related applications.

## 1. Introduction

Persimmon (*Diospyros kaki* L.) belongs to the Ebenaceae family and has become one of the most popular fruits worldwide [1]. Persimmon is believed to have originated in China more than 3000 years ago and was subsequently cultivated in Japan and the Korean Peninsula before spreading to Europe and other tropical and temperate regions [2,3,4]. According to the latest available FAOSTAT data, global persimmon production reached approximately 5.07 million tonnes in 2024, with China accounting for around 80% of total production [5]. The continued dominance of China and other major Asian producers highlights the geographical concentration and commercial importance of persimmon production [5,6]. Persimmon cultivars are typically divided into astringent and non-astringent types, as per their content of soluble tannins, which are known as proanthocyanidins (PAs), in the pulp at the time of harvest [3,7]. Astringency arises from the interaction between salivary proteins and persimmon tannins, resulting in a dry mouthfeel due to inhibition of lubrication [4]. In non-astringent varieties, such as ‘Fuyu’ and ‘Jiro’, tannins become insoluble as the fruit matures on the tree, making the fruit palatable while still firm. Conversely, at harvest astringent types like ‘Hachiya’ and ‘Rojo Brillante’ retain their soluble tannins more than non-astringent types; therefore, postharvest treatments should be applied to remove astringency, including hot-water immersion, which encourages the breakdown of tannins by triggering enzymes, and carbon dioxide or ethanol gas exposure to induce the formation of acetaldehyde and further solubilize the retained tannins [8,9].

In addition to tannin-related traits, persimmon classification can be determined by changes in pulp colour and pollination characteristics. Varieties exhibiting pollination-constant characteristics develop uniformly coloured flesh regardless of seed formation. At the same time, pollination-variant cultivars may display localized browning or pigmentation in the pulp due to oxidative processes depending on pollination success and seed development [4,10]. Based on the changes in flesh colour associated with pollination and seed development, persimmons can be divided into four types as summarized in Table 1 as (i) pollination-constant non-astringent (PCNA), (ii) pollination-constant astringent (PCA), (iii) pollination-variant non-astringent (PVNA), and (iv) pollination-variant astringent (PVA) [5,11]. In pollination-variant (PV) types, seed-derived production of acetaldehyde and ethanol is crucial in reducing astringency. Specifically, acetaldehyde induces polymerization and solubilization of the soluble tannins, which renders them less reactive and reduces astringency. In PVNA cultivars, high levels of acetaldehyde result in tannin coagulation and non-astringent flesh. However, under poor pollination conditions, acetaldehyde levels could be insufficient with few seeds, and astringency remains in the pulp. In contrast, PVA cultivars exhibit persistent astringency despite being pollination-variant, as their seeds produce inadequate quantities of acetaldehyde to trigger effective tannin solubilization [5]. PCA cultivars similarly retain astringency because the amount of acetaldehyde produced by seeds is minimal, and thus the process of tannin solubilization does not occur. In PCNA cultivars, a different mechanism is observed: tannin accumulation is ceased early during fruit development, leading to natural astringency loss on the tree independent of pollination or seed formation [5,12].

Persimmon is appreciated not only for its sensory appeal but also for its growing reputation as a functional food. Numerous studies have demonstrated the antioxidant, anti-inflammatory, antidiabetic, antihypertensive, and lipid-lowering properties of persimmon-derived constituents [1,13]. These bioactivities are largely attributed to the high content of polyphenols, flavonoids, carotenoids, vitamins, especially vitamin A and C, dietary fibre, and tannins [14]. However, most research has focused on the fruit pulp, which is often the primary edible part of the persimmon. Although the pulp remains a valuable source of bioactive compounds, recent investigations suggest that other anatomical parts, including the peels, calyxes, seeds, and leaves, are equally or more potent in terms of their phytochemical constituents [1,11]. Different maturation mechanisms lead to variations in chemical composition, highlighting their suitability for studying their functional characteristics. However, although much attention has been focused on fruit pulp, the underexplored tissues that are typically discarded during processing have emerged as rich sources of bio-functional compounds, warranting greater scientific and industrial attention.

In recent years, the issue of food loss and underutilized plant parts has become pressing [15]. In persimmon production, a substantial number of by-products are discarded or underutilized [16]. According to the research [16], up to around 30% of the total persimmon volume produced may be wasted in Spain, with 38.5% of the economic losses, referring to the number of commercialized persimmon kilograms on average. Although global statistics on persimmon waste remain limited, available evidence suggests that substantial losses occur during production and processing. This level of loss not only represents economic inefficiency, but also contributes to the accumulation of organic waste, with environmental implications such as increased greenhouse gas emissions from decomposition and unutilized agricultural inputs [16]. With the growing global emphasis on waste valorization, understanding the bioactive potential of persimmon by-products is crucial for advancing circular economy initiatives by enabling the conversion of waste materials into value-added products.

In response, increased efforts have been focused on enhancing the value of fruit by-products as part of broader circular bioeconomy strategies. Various industries extract the valuable compounds for their potential use as functional food ingredients and biodegradable materials, shifting toward sustainable food and economic systems [1,4]. Among these, persimmon by-products, including leaves, peels, seeds, and calyxes, show considerable phytochemical potential. However, current research efforts remain limited in scope, with few studies offering comprehensive comparisons across different tissues and cultivars. Due to a lack of systematic investigations, challenges for targeted product development and industrial applications have arisen. Furthermore, variation in research methods and insufficient clinical evidence limit the translation of laboratory findings into widespread use [14]. The terms functional food and nutraceutical are widely used in the scientific literature; however, they are not internationally harmonized regulatory categories, and their interpretation varies across jurisdictions. In this review, these terms are therefore used in a scientific context to describe the potential food and health-related applications of persimmon-derived bioactive compounds rather than as legally defined product categories. Accordingly, evidence of biological activity, particularly from in vitro and animal studies, should be distinguished from clinical evidence and should not be interpreted as sufficient substantiation for authorized health claims. This review aims to address these gaps by providing a comprehensive and comparative analysis of bioactive compound profiles across different persimmon by-products, particularly peel, seed, leaf, and calyx, and cultivars. We synthesize the available literature on phytochemical composition, associated biological activities, extraction approaches, and potential applications of these by-products in food, nutraceutical, pharmaceutical, and industrial contexts. The review also discusses the challenges that currently hinder the commercial valorization of these tissues. By integrating these perspectives, this review underscores the potential of persimmon by-products as underutilized resources for sustainable ingredient and product development.

## 2. Methodology

This review was reported in accordance with the Preferred Reporting Items for Systematic Reviews and Meta-Analyses (Table S1; PRISMA 2020 [17]) reporting guideline to ensure transparent reporting of the literature search and study selection process. This literature review was conducted to synthesize the available evidence on the phytochemical composition, biological activities, and industrial applications of persimmon (*D. kaki* L.) by-products. Given the broad and heterogeneous nature of the available literature, including compositional analyses, in vitro and in vivo investigations, and applied research, a structured search of the literature followed by a qualitative/narrative synthesis approach was adopted.

A comprehensive literature search was carried out across major scientific databases, including Scopus, Web of Science, PubMed, and ScienceDirect, covering publications from 2000 to March 2025. Relevant studies were identified using combinations of keywords such as persimmon, Diospyros kaki, by-products, peel, seed, leaf, calyx, phytochemicals, bioactive compounds, antioxidant, and functional food. Additional relevant studies were identified through manual screening of the reference lists of key publications. The database searches identified 512 records, comprising 198 records from Scopus, 156 from Web of Science, 89 from PubMed, and 69 from ScienceDirect. An additional 15 records were identified through screening the reference lists of relevant articles. Following the removal of 251 duplicate records, 276 records remained for title and abstract screening. Of these, 187 records were excluded because they were not relevant to the review scope. The remaining 89 articles were assessed for eligibility through full-text review, of which 53 were excluded for the reasons presented in Figure 1. Consequently, 36 studies were included in the final review. The study-selection process is illustrated in the PRISMA 2020 flow diagram (Figure 1).

### 2.1. Inclusion Criteria

Studies were considered eligible if they focused on persimmon by-products, including peels, seeds, leaves, and calyx, reporting information on phytochemical composition, bioactive compounds, biological activities, extraction methods, or potential applications in food, nutraceutical, pharmaceutical, or industrial contexts. Only peer-reviewed articles published in English were included.

### 2.2. Exclusion Criteria

Studies were excluded if their focus was exclusively on persimmon fruit pulp without relevance to by-products, were unrelated to Diospyros kaki or other persimmon species, lacked sufficient scientific detail, or were not published in peer-reviewed journals. Conference abstracts, editorials, reports, and other non-peer-reviewed publications were also excluded.

### 2.3. Study Screening and Data Extraction

The literature screening and data-extraction process was conducted by three authors. Retrieved records were initially screened based on their titles and abstracts, followed by full-text assessment of potentially relevant articles according to the predefined inclusion and exclusion criteria. The three authors reviewed the identified literature and assessed study eligibility, with decisions discussed among the authors to reach consensus. Relevant information extracted from the included studies comprised the persimmon by-product or plant part investigated, phytochemical composition, analytical and extraction methods, biological activities, and reported applications.

Because the available studies varied substantially in their objectives, experimental methodologies, investigated compounds, and outcome measures, a quantitative meta-analysis was not considered appropriate. Therefore, the findings were synthesized qualitatively and narratively and organized according to persimmon by-product type and functional properties to identify current knowledge, research gaps, and future research opportunities.

## 3. Phytochemical Composition of Persimmon Tissues

In general, persimmons are rich in a diverse range of phytochemicals with potential biological activities. An overview of the main composition in each persimmon part is presented in Figure 2. To explore the phytochemical diversity and potential functional applications of persimmons, the major compounds reported in the leaves, peels, seeds, calyx, and fruit pulp are summarized below.

### 3.1. Leaves

Recent studies have shown that persimmon leaves are a rich source of phytochemicals, particularly flavonoids and other phenolic compounds [18]. Compositional analyses have identified a diverse range of flavonoids with antioxidant activity, highlighting persimmon leaves as a potential source of natural antioxidant compounds [19]. In vitro and experimental studies have further reported that persimmon leaf extracts may influence glucose metabolism and lipid-related pathways [20]. In addition, antimicrobial and skin-protective activities have been reported in experimental studies, suggesting potential applications in food, cosmetics, and other functional products [3].

The compounds listed in Table 2 represent a chemically diverse group of phenolics, flavonoids, and triterpenoids that may contribute to the biological activities reported for persimmon leaves. However, evidence for these activities varies considerably in strength. While compositional studies establish the presence of specific phytochemicals, antioxidant, antimicrobial, metabolic, and other biological activities have predominantly been demonstrated through in vitro and animal studies [3,21]. Therefore, these findings should be considered as evidence of biological potential rather than established therapeutic effects. The limited availability of human clinical evidence remains an important limitation. Further research is therefore required to clarify compound bioavailability, structure–activity relationships, mechanisms of action, safety, and efficacy in relevant animal and human models before persimmon leaf phytochemicals can be confidently applied in functional foods or pharmaceutical products.

### 3.2. Peels

Historically, persimmon peels have been discarded during processing and considered a by-product with limited commercial value. According to a previous study [22], peels were treated as fibrous waste during the early stage of utilizing persimmons, primarily due to their tough texture and high tannin content, which contributes to the astringency and unfavourable sensory properties. However, recent phytochemical investigations have changed this perception. Compositional studies indicate that persimmon peel can contain higher concentrations of phenolic compounds and carotenoids than the pulp, including gallic acid, catechin, epicatechin, and β-cryptoxanthin [22,23].

The findings summarized in Table 2 demonstrate the potential of persimmon peel as a source of bioactive compounds for functional food and antioxidant applications. Several individual compounds identified in the peel, including gallic acid and β-cryptoxanthin, have been associated with antioxidant or other biological activities; however, the extent to which these activities occur following consumption of persimmon peel has not been established. Some in vitro studies have reported radical-scavenging, LDL-protective, and effects on cancer-cell-related pathways [22,23]. These findings therefore indicate biological potential rather than confirmed health benefits in humans. Furthermore, dietary fibre may account for up to 40% of the dry matter of persimmon peels, supporting their potential use as a fibre-rich ingredient [24]. Further in vivo and human studies are required to establish the physiological relevance of these compositional and in vitro findings and to determine the effects of processing and bioavailability.

### 3.3. Seeds

Although persimmon seeds are often neglected, they are a potentially valuable source of nutrients and phytochemicals. Recent emerging studies have revalued these persimmon by-products due to their abundant bioactive compounds (Table 2).

Compositional analyses have reported that persimmon seeds contain approximately 70.4–78.3% total fatty acids, with linoleic and oleic acids being major components [22]. Polyphenols and tannins are present in the seeds and have demonstrated antioxidant and antimicrobial activities in experimental studies [23]. Some reports have additionally suggested anticancer activity; however, these findings are primarily based on in vitro evidence and should not be interpreted as evidence of anticancer efficacy in humans. Thus, although persimmon seeds are currently underutilized, their chemical composition indicates potential for development as a functional food or ingredient. Nonetheless, current evidence is largely limited to compositional characterization and in vitro investigations. Future research should focus on extraction optimization, safety and toxicological assessment, bioavailability, and validation in appropriate animal and human studies.

### 3.4. Calyx

Persimmon calyx has received increasing research attention because of its phytochemical composition and potential functional applications. Studies have shown that calyx tissues contain considerable levels of polyphenols, including gallic acid, caffeic acid, and chlorogenic acid, as well as flavonoid and tannin derivatives [25].

The compounds listed in Table 2 have been associated with antioxidant and anti-inflammatory activities, although the strength of evidence differs among individual compounds and extracts. In vitro studies have also reported inhibitory effects of calyx extracts on the proliferation of certain cancer cell lines, with apoptosis proposed as a possible mechanism [1]. These findings provide preliminary evidence of biological activity but do not establish cancer-preventive or therapeutic effects in humans. Furthermore, the identity and abundance of individual flavonoids in persimmon calyx remain incompletely characterized, although quercetin derivatives have been proposed. Organic acids have also been suggested to occur in calyx tissues, but detailed compositional data remain limited.

Overall, persimmon calyx represents a potential source of phytochemicals for further investigation and valorization as a food or agricultural by-product. However, additional compositional, in vitro, animal, and ultimately human studies are needed to establish its biological significance, safety, and potential functional applications.

### 3.5. Fruit Pulp

Given its high nutritional profile and sensory appeal, persimmon pulp holds great promise as a functional food ingredient. The fruit itself contains 79% water, 0.7% and 0.4% pectin and protein, respectively [6]. In addition to its nutritional components, the pulp contains a range of phytochemicals and micronutrients (Table 2).

Compositional studies indicate that persimmon pulp contains natural sugars and dietary fibre, together with polyphenols, flavonoids, carotenoids, tannins, vitamins A and C, minerals such as potassium and magnesium, and amino acids [22,24]. These constituents have been associated with antioxidant and other biological activities in experimental studies. However, the presence of these compounds does not establish clinical or therapeutic effects in humans by itself. Some studies have reported potential antioxidant, anti-inflammatory, lipid-related, and metabolic effects, but the evidence should be interpreted according to the experimental model used [1,22]. Therefore, further research is required to determine how cultivar, ripening stage, processing, digestion, and bioavailability influence the phytochemical composition and biological activity of persimmon pulp and its potential use in functional food applications.

Comparison of the reported phytochemical composition indicates that persimmon peels, leaves, and calyxes can contain relatively high concentrations of phenolic compounds compared with other tissues, suggesting their potential as sources of phytochemicals for further extraction and characterization. However, higher phytochemical concentrations do not necessarily translate into greater biological or health effects, and comparative studies using standardized extraction and biological assays are required to establish their relative functional value. Moreover, direct comparison among studies remains challenging because extraction conditions vary considerably, including solvent type and concentration, solid-to-solvent ratio, extraction time, temperature, and extraction technique. Greater standardization and transparent reporting of these parameters, together with consistent analytical procedures, would improve reproducibility and allow more meaningful comparisons of phytochemical recovery and biological activity across persimmon tissues and cultivars.

## 4. Major Health Effects and Corresponding Mechanisms

### 4.1. Antioxidant Activity

The antioxidant effects of persimmon tissues, particularly leaves, peels, and seeds, are primarily attributed to polyphenols [26]. Antioxidant mechanisms are not only measurable through biochemical assays but are closely tied to the specific structural features of the molecules involved.

According to a previous study [27], phenolic compounds are present in persimmons as free and bound forms. As a result, phenols are structurally simple or highly polymerized molecules with an aromatic ring with hydroxyl substituents [28]. Among them, phenolic acids, which comprise two subgroups, including hydroxybenzoic and hydroxycinnamic acids, and flavonoids are the main dietary components [22].

Among phenolic acids, hydroxybenzoic acids such as gallic, *p*-hydroxybenzoic, protocatechuic, vanillic, and syringic acids share the same C6-C1 structure. In contrast, hydroxycinnamic acids are aromatic substances with a 3-carbon (C6–C3) side chain, such as caffeic, ferulic, *p*-coumaric, and sinapic acids (Figure 3) [29]. These structures could affect their radical-scavenging properties due to the number and position of hydroxyl groups, in relation to the carboxyl functional group [30]. Therefore, phenolic acids are known to have a structure–activity relationship [22]. Hydroxyl groups donate hydrogen atoms or electrons to radicals, converting them into stable and non-reactive species while forming resonance-stabilized antioxidant radicals.

On the other hand, flavonoids have a common C6-C3-C6 backbone composed of two phenolic rings (A and B) and a central heterocyclic ring (C) [22]. Variations in the C-ring oxidation status and hydroxylation pattern across the A and B rings give rise to distinct subclasses such as anthocyanins, flavonols, flavones, and flavan-3-ols [22]. Therefore, the degree of hydroxylation and glycosylation significantly influences electron donation capacity, radical stabilization, and bioavailability. Persimmon-derived flavonoids can act as electron donors, reducing oxidized intermediates such as Fe^3+^ to Fe^2+^, while structural features such as double-bond conjugation may contribute to stabilization of the resulting antioxidant species [31], suggesting that their structural features, such as a carbon double bond, would enhance electron conjugation, which stabilizes the resulting antioxidant radical and facilitates electron donation [22,32]. Meanwhile, flavonoids and PA can chelate Fe^2+^ to inhibit Fe^2+^-ferrozine complex formation, thereby reducing hydroxyl radical formation [31]. Therefore, the hydroxyl and carbonyl groups on the flavonoid skeleton that form stable complexes with Fe^2+^ ions could provide secondary antioxidant protection [21,33].

### 4.2. Hypolipidemic Activity

The hypolipidemic effects of persimmon-derived phytochemicals are increasingly recognized, particularly in peels and leaves rich in soluble fibre, flavonoids, tannins, and other polyphenolic compounds. These effects are mediated through multiple biochemical and physiological mechanisms, including regulation of lipid profile, inactivation and inhibition of lipogenic pathways, and regulation of gut microbiota [33].

Dietary fibre and tannins in persimmon contribute primarily to limiting intestinal lipid absorption. These compounds interact with dietary cholesterol and bile acids in the gastrointestinal tract, forming complexes that make them resistant to reabsorption [34]. Consequently, the excretion of bile acids is increased, which stimulates hepatic cholesterol catabolism to replenish bile pools as presented in Figure 4 [33]. As a result, fecal excretion is enhanced; circulating LDL-cholesterol levels and lipid absorption are reduced [35].

Second, flavonoids and polyphenols in persimmon tissues influence lipid metabolism directly at the molecular level. For example, tannins significantly (*p* < 0.05) inhibit the expression of key lipogenic enzymes and transcription factors, such as acetyl-CoA car-boxylase (ACC), fatty acid synthase (FAS), sterol regulatory element binding protein 1 (SREBP1), and stearoyl-coenzyme A desaturase 1 (SCD1) [36]. Simultaneously, tannins in persimmons enhance the expression of fatty acid oxidation regulators, which is mediated by the activation of AMP-activated protein kinase (AMPK) and suppression of specific microRNAs (miRNAs) [36,37], reducing the accumulation of hepatic triglycerides [33].

Finally, these effects may be driven by modulation of gut microbiota; specifically, persimmon tannins increase the abundance of Bacteroidetes and suppress Firmicutes [38], avoiding dysbiosis in microbial composition [33]. Moreover, persimmon tannins promote the growth of beneficial bacteria such as *Bifidobacterium* sp. and *Lactobacillus* sp. while inhibiting harmful species like *Escherichia coli* and *Enterococcus* sp. [38]. This dysbiotic shift increases the production of short-chain fatty acid (SCFA), suggesting an improved lipid metabolism [38].

**Figure 4:**
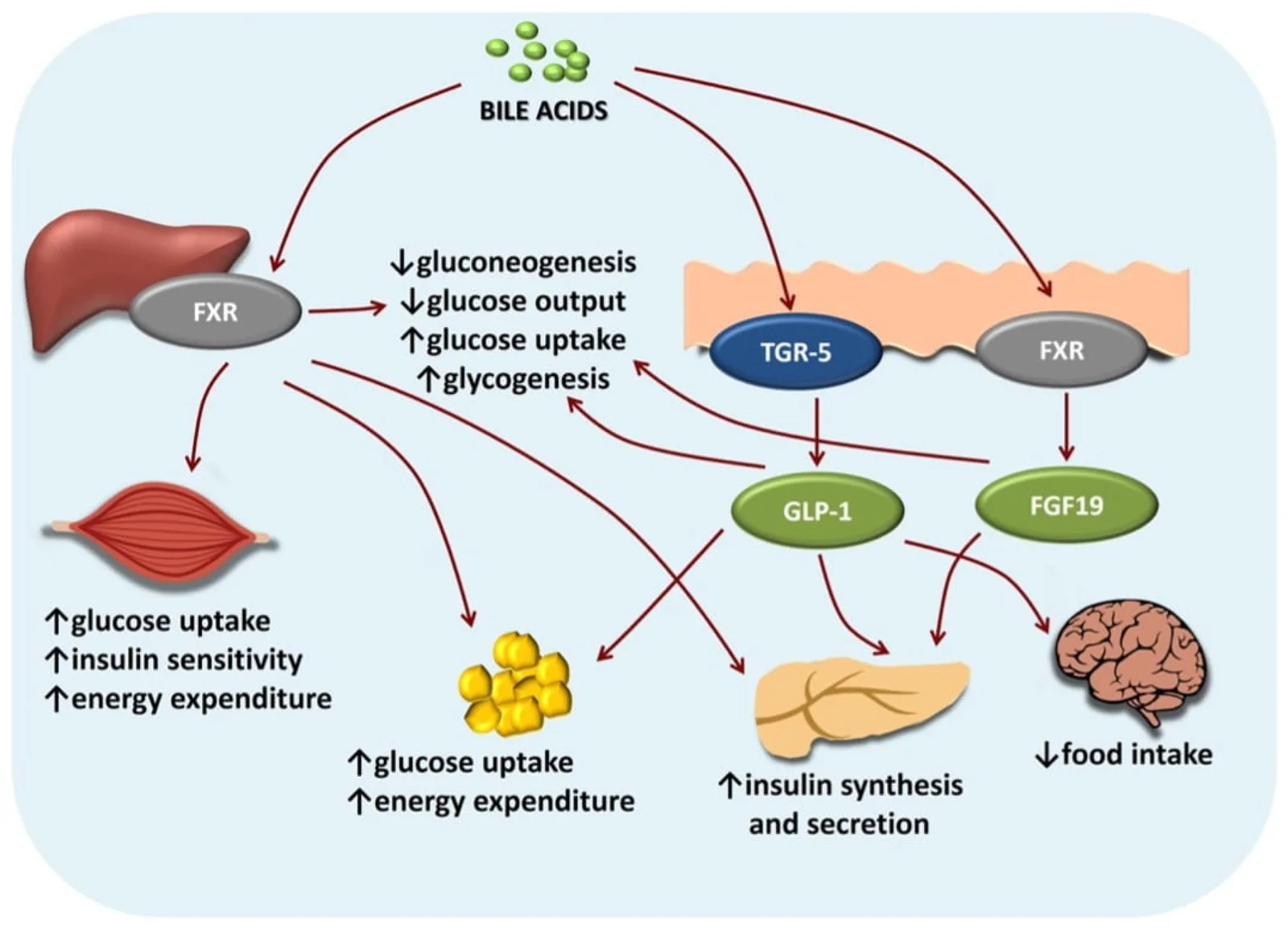
The mechanism of bile acid regulating lipid metabolism [39]; ↑ Increase; ↓ decrease.

### 4.3. Anti-Inflammatory Activity

Inflammation is a physiological response to external stimuli and is closely associated with the development and progression of numerous diseases [40], including inflammatory bowel disease (IBD) and cancer [33]. Therefore, modulation of excessive or persistent inflammatory responses represents an important area of research.

Polyphenolic compounds in persimmon, particularly tannins present in the leaves and fruits, have been investigated for their potential anti-inflammatory activities. Dysbiosis characterized by an increased abundance of Proteobacteria and reduced Actinobacteria has been implicated in the pathogenesis of IBD [41]. Persimmon-derived bioactive compounds have also been investigated for their potential to modulate gut microbiota and support gut health. Persimmon tannins have demonstrated anti-inflammatory activity through modulation of inflammatory pathways, including NF-κB signalling, together with changes in gut microbiota composition [33,41]. However, these findings are predominantly based on experimental studies and require further confirmation in humans.

In addition to their potential anti-inflammatory effects, persimmon-derived bioactive compounds have been investigated for cancer-related biological activities, which may be associated with constituents such as carotenoids and flavonoids [22,32,42]. These compounds have been associated with cellular protection and the regulation of cell growth, differentiation, and apoptosis [22,43]. Experimental studies have reported that persimmon-derived bioactive compounds can inhibit cell proliferation and induce apoptosis in several human cancer cell lines [42,44]. However, these findings are predominantly based on in vitro evidence and should not be interpreted as evidence of anticancer efficacy in humans.

### 4.4. Antidiabetic Activity

On the other hand, persimmon leaves and peels have shown substantial capacity as sources of natural antidiabetic compounds. These effects are mediated through multiple complementary mechanisms, primarily including the inhibition of carbohydrate-digesting enzymes such as α-glucosidase and α-amylase, an increase in glucose uptake by the cells, regulation of insulin sensitivity, and antioxidant protection, by the bioactive constituents, including flavonoids, condensed tannins, gallic acid, and catechin derivatives [45].

Aqueous methanolic extracts of persimmon leaves have demonstrated noncompetitive inhibition of α-glucosidase from both baker’s yeast and rat intestine, with IC_50_ values of 1.22 µg/mL and 32 µg/mL, respectively. These inhibitory effects were stronger than those observed for acarbose, which was used as the pharmaceutical comparator [46]. The research also validated the functional relevance of enzyme inhibition through in vivo studies in both normoglycemic and streptozotocin-induced diabetic rats [46]. In this study, after consuming glucose and maltose, the groups treated with extracts of persimmon leaves at 250 to 1000 mg/kg body weight showed a reduction in blood glucose levels in a dose-dependent manner, in which the highest dose (1000 mg/kg of bw) produced effects comparable to 50 mg/kg bw of acarbose after 120 min, offering a significant hypoglycemic effect [46].

The evidence supporting α-glucosidase inhibition suggests that this may contribute to the antihyperglycemic effects of persimmon leaves. In addition, persimmon leaf supplementation may improve insulin sensitivity and hepatic glucose metabolism. In an animal study, persimmon leaf supplementation significantly reduced fasting blood glucose and HOMA-IR without altering insulin levels over a 5-week feeding period, suggesting improved insulin sensitivity [47]. Mechanistically, persimmon leaves increased both the activity and gene expression of glucokinase, a key enzyme involved in glucose phosphorylation and subsequent storage and glycolysis, and simultaneously increased hepatic glycogen content, thereby promoting hepatic glucose utilization [47].

In parallel, persimmon leaves have been reported to reduce the activities of key gluconeogenic enzymes, including glucose-6-phosphatase (G6Pase) and phosphoenolpyruvate carboxykinase (PEPCK), but without corresponding changes in their gene expression [47,48]. These findings indicate that the suppression of hepatic gluconeogenic capacity occurs at the enzyme-activity level rather than through transcriptional regulation. PEPCK gene transcription is known to be subject to complex hormonal regulation [49]. The reduced activities of G6Pase and PEPCK may therefore reflect post-transcriptional or post-translational regulation, altered enzyme function, or other metabolic mechanisms; however, the precise mechanism remains to be determined. Together, increased glucokinase activity and reduced G6Pase and PEPCK activities would be expected to favour hepatic glucose utilization and reduce endogenous glucose production, thereby potentially improving glycemic control.

More broadly, the recovery of phenolic compounds from plant-derived by-products and their subsequent incorporation into food systems represents a practical strategy for converting underutilized biomass into value-added functional ingredients [50].

Kinetic analyses have also indicated noncompetitive inhibition, suggesting that the extract may bind to an allosteric site rather than competing directly with the substrate [46]. As a result, a more consistent inhibition of dietary sugar levels would be provided by persimmon leaves and peels [46]. In contrast, although the polymers of PA from persimmons were suggested as one of the effective α-amylase inhibitors, the inhibition activity tends to be weaker and variable, but they still contribute to the cumulative suppression of carbohydrate digestion when incorporated with α-glucosidase inhibitors [51].

### 4.5. Antimicrobial Activity

In recent years, persimmons have been the focus of attention for selective antimicrobial potential, mediated through their high content of tannins and phenolic compounds [52]. These properties enable them to inhibit a broad range of Gram-positive and Gram-negative bacteria, including foodborne pathogens and intestinal opportunists, such as *Staphylococcus aureus*, *Staphylococcus epidermidis*, *Escherichia coli, Salmonella* sp., and *Pseudomonas aeruginosa* [53,54].

In fact, antimicrobial activity of persimmons is regulated by the generation of hydrogen peroxide, which may be driven by the inhibition of DNA gyrase and nucleic acid synthesis, pore formation, and alteration of the cell membrane or cell walls [52,53]. Among all the tested parts in the research [55], the leaf tissues were shown to consistently produce the highest levels of hydrogen peroxide, aligning with their superior antimicrobial efficacy. For example, leaf extract completely suppressed the growth of *Campylobacter sputorum*, *Bacteroides thetaiotaomicron*, and *S. aureus*, while stem extracts showed intermediate inhibition [55]. They discovered that persimmon leaf extracts strongly inhibit *E. coli* and *S. aureus*, with over 95% of growth inhibition achieved at extract concentrations of 4.8–5.7 × 10^−4^ mol/L hydrogen peroxide. By treating with catalase, the antibacterial effect was neutralized, confirming that reactive oxygen species were the primary inhibitory agent [55]. This mechanism is consistent with prior studies showing the generation of reactive oxygen species derived from catechin in tannin-rich plants. Notably, the extracts have exhibited minimal effects on beneficial bacteria such as *Lactobacillus casei*, *Bifidobacterium longum*, and *Enterococcus faecalis*, which are known to be less sensitive to hydrogen peroxide, suggesting a degree of microbial selectivity that may benefit the balance of gut microbiota [55].

Other studies further verified that ethanolic and methanolic extracts of persimmon leaves and peels inhibit common pathogens such as *Salmonella* sp. and *Bacillus cereus*, with activity positively correlated to total phenolic content and flavonoid levels [53,56].

These findings support the development of natural antimicrobial agents from processing by-products of persimmon. However, variability in experimental results with differences in cultivar, extraction method, and microbial target suggests that studies on standardization and formulation are necessary to ensure efficacy and stability [52].

## 5. Cultivar-Specific Functional Variability

Significant variation exists among persimmon cultivars in their phytochemical profiles and associated biological activities, primarily influenced by the type of cultivar that is modulated by environmental conditions. Broadly, cultivars are classified as astringent or non-astringent, each exhibiting distinct bioactive characteristics. Astringent types such as ‘Hachiya’ accumulate high levels of soluble tannins and PA during early development, contributing to their characteristic astringency and strong antioxidant capacity [4]. They are more abundant in phenolic acids, including protocatechuic, gallic, caffeic, and ferulic acids, enhancing radical-scavenging and lipid peroxidation inhibition [4]. Consequently, extracts from astringent cultivars often show superior performance in antioxidant assays such as DPPH, ABTS, and FRAP [57].

In contrast, non-astringent cultivars such as ‘Fuyu’ and ‘Jiro’, which dominate commercial markets, undergo early tannin solubilization and accumulate more carotenoids and flavonoids. These compounds contribute to milder but more stable antioxidant profiles and superior sensory quality [4]. Differences also extend to fruit composition: astringent types show higher total soluble solids (~18.23 °B) than non-astringent types (~16.16 °B) [4], while malic acid content varies markedly between cultivars. For example, the malic acid content in ‘Hachiya’ is about 6.40 µg/mg, while it is around 11.50 µg/mg in ‘Fuyu’, which would directly affect the overall flavour and organoleptic properties [58]. Beyond fruits, leaf tissues also reflect cultivar-dependent variation. PCA-type cultivars such as ‘Bull Heart’ and ‘Diamond Bull Heart’ exhibit higher levels of polyphenols, flavonoids, and condensed tannins, which contribute to their strong antioxidant performance [6].

Cultivar variation may also be associated with geographical origin, with differences in soluble tannin, sugar, and phenolic composition reported across persimmon genotypes and origins [59]. For example, soluble tannin and sugar content in persimmons have been shown to significantly correlate with the genotype and origins, while the accumulation of phenolic compounds and carotenoids is influenced by temperature, altitude, and light exposure [57,59]. The differences in flavonoid accumulation among cultivars grown under the same environmental conditions are stated to link them with strong genetic control, whereas seasonal variation was more evident in modulating the total phenolic content and antioxidant capacity. Therefore, the interaction between cultivar genotype and environment is critical for understanding the stability and enhancement of functional properties during breeding and cultivation.

Although differences in phytochemical composition and antioxidant activity among cultivars are well documented, systematic multi-environment trials capturing both genotypic and environmental effects are rare, and few studies compare multiple plant tissues across cultivars under controlled environmental gradients. As a result, the lack of standardization in tissue maturity, extraction methods, and antioxidant testing protocols obstructs cross-study comparability. Moreover, the marked differences in phytochemical composition among persimmon cultivars are likely to affect the usability of their extracts in functional applications. As evidenced, astringent cultivars tend to accumulate higher levels of soluble tannins and condensed PA that contribute to the strong antioxidant, anti-inflammatory, and antilipidemic properties [4]. In contrast, non-astringent cultivars are generally richer in carotenoids and flavonoids, offering alternative antioxidants and sensory advantages [4]. These differences not only influence extraction yields and bioactivity but also determine the compatibility of formulation, stability of extract, and end-use efficacy. In this context, comparative data on astringent varieties serve as biochemical benchmarks for interpreting the compositional and functional patterns of non-astringent cultivars. Given the industrial prevalence of non-astringent types, future research should prioritize their tissue-specific phytochemical characterization under different environmental conditions, supported by standardized bioassays to enable consistent functional food development and by-product valorization.

## 6. Industrial and Applied Potential

### 6.1. Functional Food Development

In functional food systems, persimmons have demonstrated potential of strong application across multiple categories as summarized in Table 3 [60]. The incorporation of persimmon flour into pasta products has been investigated for its effects on phenolic composition and starch digestibility [61]. Related studies using barley- and sorghum-derived ingredients in pasta products have also reported changes in phenolic composition and antioxidant properties [62,63]. These formulations also improved the product colour and consumer acceptability and can spare the need of synthetic additives.

High-pressure processing has also been investigated as a non-thermal preservation approach for persimmon purée, with particular attention to its quality and stability during chilled storage [64]. Persimmon has also been explored for the development of value-added products, including fermented products such as vinegar, reflecting broader opportunities for its utilization in food product development [65,66].

From a technological standpoint, persimmon extracts exhibit favourable formulation properties, including enhanced functional availability and improved stability within food formulations, underscoring their strong potential for functional food development. High-pressure treatment has also been investigated for its effects on carotenoid composition and radical-scavenging activity in persimmon fruit purées [67]. Green extraction technologies have been investigated for the recovery of dietary fibre and antioxidant compounds from persimmon by-products for potential use in functional beverage development [68]. In addition, persimmon and its derived ingredients have been explored for the development of value-added food products, highlighting opportunities for their broader utilization in food formulations [66].

### 6.2. Potential Health-Related and Pharmaceutical Applications

Persimmon and its by-products are abundant sources of bioactive phytochemicals with potential applications in functional foods and other health-related products, as summarized in Table 3. Persimmon by-products are rich in dietary fibre and polyphenolic compounds, which have attracted interest for their potential applications in functional foods and metabolic health; however, further studies are required to establish their efficacy in humans [60,69,70]. Persimmon by-products have also been investigated as sustainable sources of dietary fibre and antioxidants for functional beverage development [68]. Therefore, the fibre–polyphenol matrix may have potential for the development of functional food and dietary supplement formulations, although further evidence is required to establish its health effects in humans.

Green extraction approaches have been explored for the recovery and valorisation of bioactive components from persimmon by-products [68]. The recovered components may potentially be incorporated into different food or supplement formulations; however, their stability, bioavailability, safety, and efficacy require further investigation. Different drying methods have been investigated for persimmon processing, with their effects on total bioactive compounds, phenolic profiles, and the in vitro bioaccessibility of phenolics varying according to the drying conditions applied [71].

**Table 3:** Potential food and health-related applications of persimmon-derived ingredients.

Application Category	Application Potential	Functions	References
**Bakery Products**	Flour additives	Increase bound phenolics; increase antioxidant activity; improve colour/acceptance	[61]
**Persimmon Purée**	High-pressure processing	Chilled-storage stability	[64]
**Fermented Beverages**	Persimmon vinegar	Potential application in fermented and value-added food products	[65,66]
**Persimmon fruit purée**	High-pressure treatment	Effects on carotenoid composition and radical-scavenging activity	[67]
**Health-related products**	Polyphenol- and fibre-rich ingredients	Potential applications in functional foods and health-related products	[68,69]
**Drying Technologies**	Different drying methods	Effects on bioactive compounds, phenolic profile, and phenolic bioaccessibility	[71]

## 7. Advantages and Disadvantages

Persimmons have gained increasing attention as a source of bioactive phytochemicals for the potential development of innovative food ingredients, food supplements, and other health-related products. The diverse tissues offer antioxidant, anti-inflammatory, antidiabetic, and hypolipidemic properties, supporting persimmon’s growing role in health-promoting product development. However, challenges related to extraction efficiency, stability, sensory quality, and regulatory compliance are continuing to limit its full industrial utilization. This section critically evaluates the advantages and limitations of persimmons and its relevant products from an applied science perspective.

### 7.1. Advantages

#### 7.1.1. Bioactivity and Sustainability

Persimmon tissues, particularly peel, leaves, and calyx, contain polyphenols, flavonoids, carotenoids, tannins, and dietary fibre, and extracts from these tissues have demonstrated antioxidant and other biological activities in experimental studies [1,4].

Persimmon extracts show functional activities as well. The presence of pectin and soluble fibres in the by-products of persimmon contributes to the modulation of viscosity and gelation [72]. In other words, the natural emulsifying capacity of pectin helps to form a thickened elasticity viscoelastic layer at the oil–water interface, reducing the risks of phase separation, establishing strong resistance to deformation during preparation, transportation, and storage [73]. Altogether, these functional traits position the persimmon materials as viable natural additives in various industrial food products.

Generally, the non-edible tissues of persimmons, peel, calyx, and seeds are often discarded during commercial processing, while their conversion into functional extracts or natural ingredients represents an effective sustainable bioeconomy strategy. These residues are rich in phytochemical compounds, which can be extracted and applied in food preservation, functional materials, or wastewater treatment, reducing waste, and adding economic value [1]. For example, tannin-rich peels have been used to produce antioxidant-enriched coatings or sorbents for heavy metals, suggesting a practical application for cleaning up metallic pollution [74].

Beyond product development, such valorization reduces postharvest loss, lowers carbon emissions from organic waste decomposition, and minimizes the need for synthetic additives. Valorisation of persimmon by-products may contribute to improved resource utilization and the recovery of value from materials that would otherwise be discarded [75]. In addition, reducing postharvest losses may help conserve associated land, nutrient, and economic resources, supporting more sustainable persimmon production systems [76].

#### 7.1.2. Cultivar Diversity

The diversity among persimmon cultivars enhances the functional targeting. Astringent cultivars such as ‘Hachiya’ and ‘Rojo Brillante’ are rich in soluble tannins and PA, while non-astringent cultivars such as ‘Fuyu’ contain higher carotenoid and flavonoid levels [4]. This compositional diversity may support different potential applications of persimmon-derived ingredients. Carotenoids are widely investigated for their biological activities [77], while tannin-rich fibre from persimmon fruit has been reported to reduce cholesterol levels in humans [78].

At the same time, the commercial non-astringent cultivars have gained popularity in both consumers and farmers due to their sweetness and the adaptability to a wide range of climatic conditions, allowing international trade and the development of the persimmon-based products [2]. Nevertheless, although astringent cultivars are less suitable for various growing conditions, they are largely preferred by local consumers given by the treatments to remove astringency that is controlled after harvest [2]. As a final point, the agronomic diversity among persimmon cultivars enables us to target functional applications and market expansion, supporting both industrial innovation and consumer preferences.

### 7.2. Disadvantages

#### 7.2.1. Processing Limitations

During the processing of persimmon, the choice of solvent, pH sensitivity, and processing conditions affect the bioactive stability and recovery yields. The recovery of bioactive compounds from persimmon by-products is influenced by the extraction approach and processing conditions [68]. Although some advanced methods of ultrasound-assisted extraction (UAE), NADES, and supercritical CO_2_ can improve the recovery, but they are associated with higher costs, specialized equipment, and lower scalability for industrial use. Bioactive compounds may also be affected by processing and storage conditions, with polyphenols particularly susceptible to temperature-related changes [22,60,79], that will further reduce the efficiency of processing along with the supply chain. Postharvest treatments, including high-CO_2_ exposure, may also be used to reduce soluble tannins and remove astringency in astringent cultivars [80]. Given these points, without significant agronomic investment, the limitations in handling would not only add more pressure on costs and infrastructure but also limit the cultivar expansion to non-native regions [2].

#### 7.2.2. Application Constraints

The diverse phytochemical composition among persimmon cultivars presents both opportunities and challenges for their functional application. Differences in bioactive composition and processing conditions can influence the characteristics and antioxidant properties of persimmon-derived materials [81,82]. In particular, tannins may influence sensory acceptability through their interactions with salivary proteins, which contribute to the perception of astringency [83,84]. Such compositional and sensory variability should therefore be considered when developing persimmon-derived ingredients for food applications.

Moreover, the insufficient understanding of cultivar classification and extraction protocols further complicates industrial reproducibility and hinders scalable formulation strategies [1,85]. These limitations indicate that the reported biological activities should not be directly equated with the already established efficacy of persimmon-derived products in humans; therefore, further standardization and validation are required before broader functional or nutraceutical applications can be substantiated. While non-astringent cultivars have gained success in fresh markets due to their sweetness and transportability, the functional valorization of astringent types remains underdeveloped; consequently, international trade is limited, obstructing the circular economy’s profitable expansion [2]. A further limitation is the scarcity of human clinical evidence supporting the biological effects reported for persimmon by-products. Most available studies are based on compositional analysis, in vitro assays, or animal models, which are valuable for identifying biological potential but cannot establish efficacy in humans. Well-designed human studies are therefore needed to determine bioavailability, effective intake levels, safety, and clinically relevant outcomes before these findings can be translated into health-related applications. Overall, the inconsistent biochemical profiles across tissues, genotypes, and growing conditions create major formulation and marketing challenges for persimmon-derived products, especially in the global functional food and nutraceutical sectors. Therefore, to enhance the functionality and commercial viability of persimmon-derived products, addressing these challenges through optimized approaches is required.

## 8. Future Directions

The processing and application challenges highlight the important research gaps. The issues mentioned previously suggest a need for improved extraction technologies, cultivar-specific protocols, and formulation strategies. To fully understand the industrial and functional potential of persimmon-derived products, further development is required.

### 8.1. Biotechnological Innovations

Advanced extraction technologies should be prioritized to address the limitations associated with cultivar variability and compound instability. Biotechnological tools such as computational calculations for solvents, thermal dynamic predictive modelling, and AI-assisted process optimization could be used to design a selective and efficient system for extraction to specific phytochemical targets. Enzyme-assisted extraction and membrane separation techniques also offer greener and more precise alternatives compared to conventional solvent-based methods, particularly for sensitive compounds like PA or carotenoid esters [81,82]. These innovations are significant for increasing extraction yield, maintaining compound integrity, and supporting the constitution of functional products from the raw material.

### 8.2. Sustainable Production

Beyond extraction efficiency, practices of reducing waste, energy use, and environmental burden throughout the production chain should be applied to promote sustainability in persimmon processing. One key strategy is integrating all fruit tissues into product development, including peel, calyx, pulp residues, and seeds. For example, peel and calyx could be used to produce antioxidants, functional powders, or biodegradable packaging films, while seeds may serve as sources of oil [1].

Secondly, implementing green extraction systems is also important. This includes switching from petroleum-based solvents to bio-based solvents, using low-temperature or ultrasound-assisted extraction to conserve energy [86], and incorporating closed-loop solvent recovery to minimize emissions and hazardous waste [87]. By introducing these practices into the design of persimmon processing facilities, the industry may shift toward a low-impact, resource-efficient approach which encourages global sustainability goals and supports persimmons as a functional crop.

### 8.3. Building Capacity

Due to the need for technical innovations, farmer participation is critical. However, in many persimmon-producing regions, especially across Asia, growers lack access to training on cultivar selection, postharvest and pre-processing management [88]. Therefore, farmers might be guided in identifying high-value cultivars for particular industrial applications by operations that connect functional trait expression, such as soluble tannin levels or carotenoid density, with agronomic knowledge [89]. Consequently, it will allow farmers to engage in higher-value marketplaces in addition to improving the overall quality of persimmon fruit.

### 8.4. Economic Support

Finally, future work needs to focus on integrating persimmon-based ingredients into the global bioeconomy. With rising consumer demand for green antioxidants and metabolic health products, there is huge potential in persimmons. Unlocking the broader utilization of persimmon products necessitates ensuring extract quality, standardization, and functional efficacy across both production batches and cultivars. Therefore, establishing regulatory permission, promoting clinical validation, and developing quality assurance procedures to ensure accreditation, certification, and inspection should be critical to expand international commerce and market entry into the functional and nutraceutical industries [90,91].

## 9. Conclusions

In conclusion, persimmon by-products, including peel, seed, leaf, and calyx, represent promising sources of phytochemicals such as polyphenols, flavonoids, carotenoids, and condensed tannins. The available literature indicates that these tissues possess a range of reported biological activities; however, the evidence is predominantly derived from in vitro and animal studies and therefore should not be interpreted as definitive evidence of clinical efficacy in humans. Their diverse phytochemical profiles across tissues and cultivars provide opportunities for value-added ingredient development, but substantial variability in composition, extraction procedures, and experimental approaches remains a challenge for reproducibility and application. Future research should therefore prioritize standardized extraction and analytical methods, characterization of structure–activity relationships, assessment of safety and bioavailability, and well-designed in vivo and clinical studies to validate the reported biological effects. Product-level studies are also required to establish stability, functionality, sensory acceptability, and scalability in food products, food supplements, and other health-related applications. In parallel, sustainable processing and whole-tissue utilization may contribute to improved resource efficiency and waste valorization. Thus, while persimmon by-products have considerable potential as bioactive ingredients and for circular bioeconomy applications, their health-promoting and commercial benefits require further validation before definitive claims can be established.

## Figures and Tables

**Figure 1:**
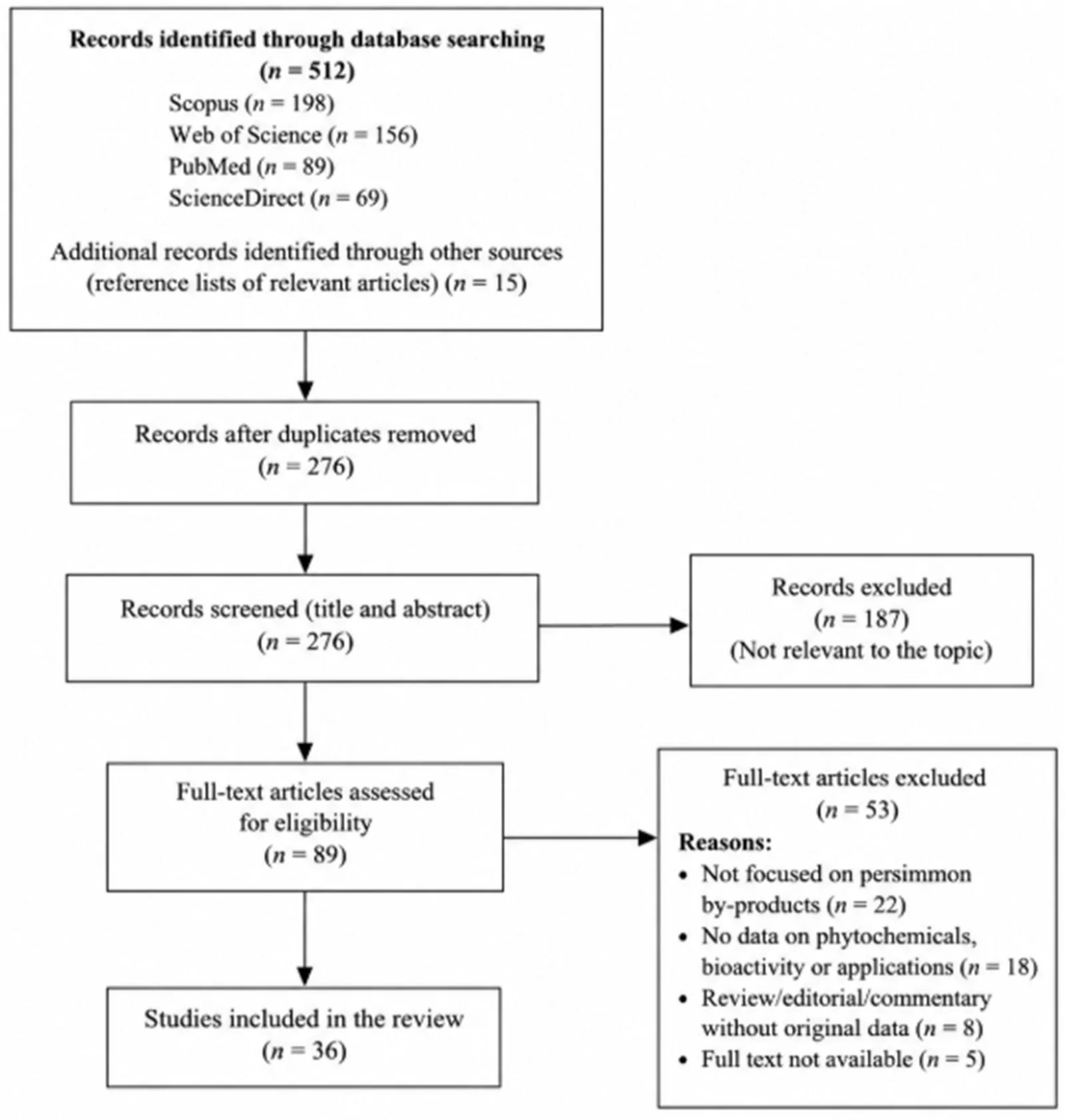
PRISMA 2020 flow diagram showing the identification, screening, eligibility assessment, and inclusion of studies in the review.

**Figure 2:**
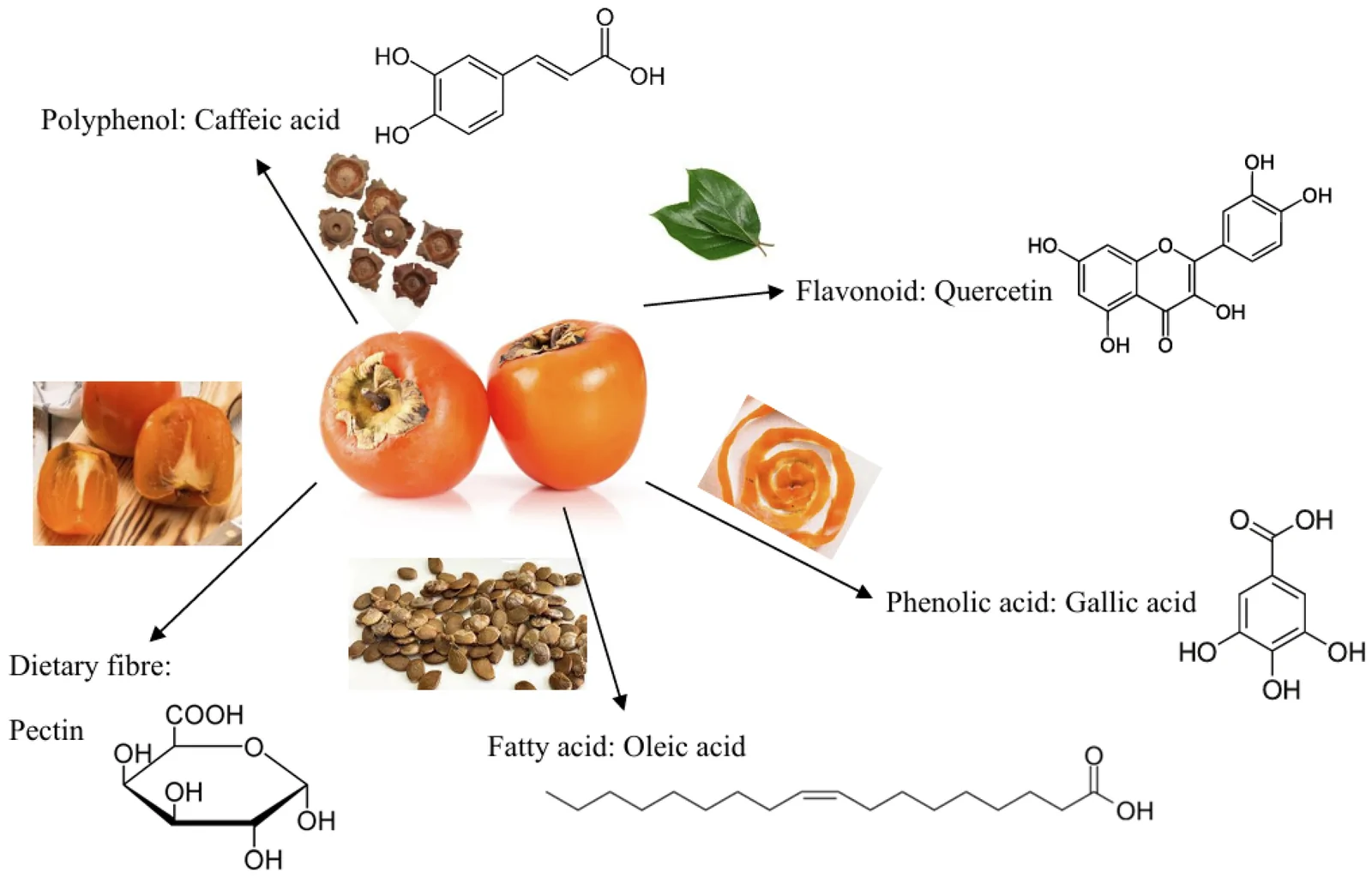
Some of the phytochemical compositions of each persimmon part.

**Figure 3:**
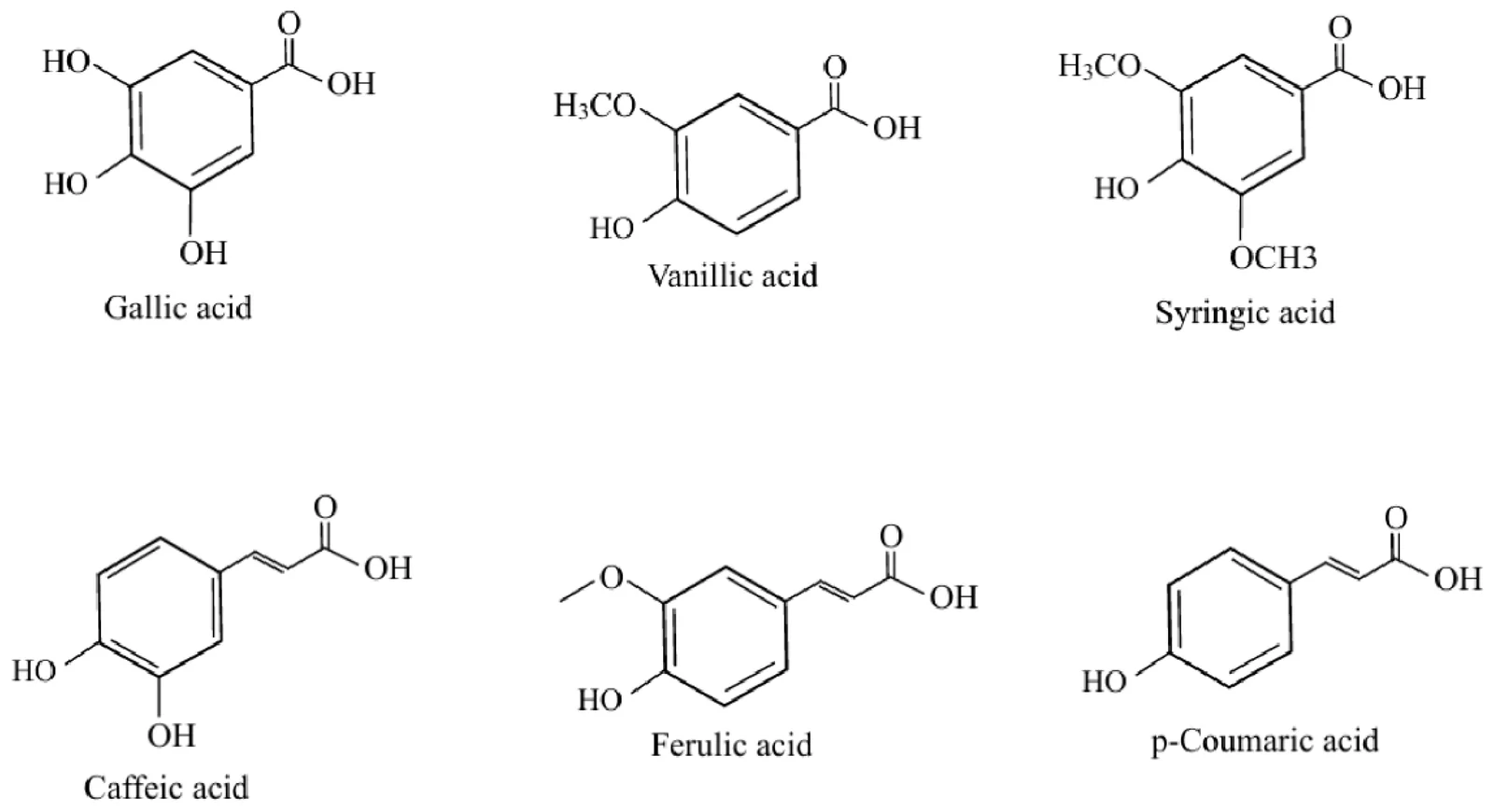
Chemical structures of representative hydroxybenzoic acids (gallic acid, vanillic acid, and syringic acid) and hydroxycinnamic acids (caffeic acid, ferulic acid, and *p*-coumaric acid).

**Table 1:** Horticultural classification of persimmon cultivars by astringency and flesh colour of fruit [12].

Seed Effect	NA (Non-Astringent)	A (Astringent)
PC (pollination constant)	PCNA Maturity: Non-astringent whether seeded or not. Flesh: Colour unaffected by seeds. Chemistry: Tannins not coagulated by ethanol when immature (VIG *).	PCA Maturity: Astringent unless treated. Flesh: Color unaffected by seeds. Chemistry: Tannins coagulated by ethanol even when immature (VDG **).
PV (pollination variant)	PVNA Maturity: Non-astringent only if seeded. Flesh: Turns brown at maturity if seeded. Chemistry: Tannins coagulated by ethanol even when immature (VDG).	PVA Maturity: Astringent unless treated. Flesh: Brown colour only around the seeds. Chemistry: Tannins coagulated by ethanol even when immature (VDG).

* VIG: Volatile Independent Group; ** VDG: Volatile Dependent Group.

**Table 2:** Phytochemical composition, extraction methods, and biological activities of persimmon by-products.

Tissue	Major Compounds	Representative Compounds	Extraction/Method	Experimental Model	Key Biological Activities	Key Findings	References
**Leaves**	Flavonoids, triterpenoids, polysaccharides, vitamin C	Quercetin, kaempferol, ursolic acid, oleanolic acid	Aqueous/methanolic extraction; HPLC analysis	In vitro, in vivo (animal)	Antioxidant, antidiabetic, anti-inflammatory	Strong α-glucosidase inhibition; improved glucose metabolism and lipid regulation	[6,18,19,20]
**Peel**	Phenolic acids, flavonoids, carotenoids, tannins, dietary fibre	Gallic acid, catechin, epicatechin, β-cryptoxanthin	Solvent extraction; spectrophotometric assays	In vitro	Antioxidant, antimicrobial, hypolipidemic	Higher phenolic content than pulp; strong radical-scavenging and LDL protection	[22,23,24]
**Seed**	Fatty acids, polyphenols, tannins, sterols, proteins	Linoleic acid, oleic acid, catechin derivatives	Lipid extraction; GC-MS	In vitro	Antioxidant, cardioprotective, antimicrobial	High unsaturated fatty acids; potential lipid-lowering and antimicrobial effects	[22,23]
**Calyx**	Polyphenols, flavonoids, tannins	Gallic acid, caffeic acid, chlorogenic acid	Solvent extraction; LC-MS	In vitro	Antioxidant, anti-inflammatory, anticancer	Induces apoptosis in tumour cell models; strong phenolic activity	[1,25]
**Persimmon tissues**	Phenolic compounds, flavonoids, tannins, carotenoids	Gallic acid, catechins, carotenoids	NADES; conventional extraction and compositional analysis	Review of experimental evidence	Antioxidant and potential health-related activities	Persimmon tissues contain diverse bioactive phytochemicals associated with antioxidant and other biological activities	[21]
**Multiple tissues**	Mixed phytochemicals (polyphenols + fibre matrix)	Phenolic acids, flavonoids, dietary fibre	Functional food incorporation	In vivo (limited), food systems	Hypolipidemic, gut health	Reduced LDL, improved lipid metabolism and gut microbiota modulation	[22]

## Data Availability

No new data were created or analyzed in this study. Data sharing is not applicable to this article.

## Supplementary Materials

The following supporting information can be downloaded at: https://www.mdpi.com/article/10.3390/nu18193194/s1, Table S1: PRISMA 2020 Checklist [17].

## References

[1] Direito R., Rocha J., Sepodes B., Eduardo-Figueira M. (2021). From *Diospyros kaki* L. (Persimmon) Phytochemical Profile and Health Impact to New Product Perspectives and Waste Valorization. Nutrients.

[2] Llácer G., Badenes M.L., Bellini E., Giordani E. (2002). Persimmon Production and Market.

[3] Jia Y., Wang Z., Liang X., Tu C., Khalifa I., Wang C., Zhu Y., Chen H., Hu L., Li C. (2024). Unlocking the potential of persimmons: A comprehensive review on emerging technologies for post-harvest challenges, processing innovations, and prospective applications. Food Chem..

[4] Murali P., Hamid N., Shams R., Dar A.H. (2023). Insights on nutritional profile, nutraceutical components, pharmacological potential, and trending utilization of persimmon cultivars: A review. Food Chem. Adv..

[5] Food and Agriculture Organization of the United Nations (FAO) (2025). FAOSTAT: Crops and Livestock Products.

[6] Zhang Y., Zhang J., Xu J., Su W., Li Z., Zhang Z. (2024). Comparative transcriptome analysis of persimmon somatic mutants (*Diospyros kaki*) identifies regulatory networks for fruit maturation and size. Front. Plant Sci..

[7] Favaretto D.P.C., Rempel A., Lanzini J.R., Silva A.C.M., Lazzari T., Barbizan L.D., Brião V.B., Colla L.M., Treichel H. (2023). Fruit residues as biomass for bioethanol production using enzymatic hydrolysis as pretreatment. World J. Microbiol. Biotechnol..

[8] Tessmer M.A., Besada C., Hernando I., Appezzato-da-Glória B., Quiles A., Salvador A. (2016). Microstructural changes while persimmon fruits mature and ripen. Comparison between astringent and non-astringent cultivars. Postharvest Biol. Technol..

[9] Besada C., Salvador A., Mir S., Shah M., Mir M. (2018). Postharvest Biology and Technology of Persimmon.

[10] Woolf A., Yahia E.M. (2011). Persimmon (Diospyros kaki L.).

[11] Hossain A., Shahidi F. (2023). Persimmon Leaves: Nutritional, Pharmaceutical, and Industrial Potential—A Review. Plants.

[12] Yonemori K., Sugiura A., Yamada M. (2000). Persimmon genetics and breeding. Plant Breed. Rev..

[13] Lee J.S., Lee M.K., Ha T.Y., Bok S.H., Park H.M., Jeong K.S., Woo M.N., Do G.M., Yeo J.Y., Choi M.S. (2006). Supplementation of whole persimmon leaf improves lipid profiles and suppresses body weight gain in rats fed high-fat diet. Food Chem. Toxicol..

[14] MGonzález C., Hernando I., Moraga G. (2021). In Vitro and In Vivo Digestion of Persimmon and Derived Products: A Review. Foods.

[15] FAO (2012). Food Wastage Footprint an Environmental Accounting of Food Loss and Waste Concept Note.

[16] Fernandez-Zamudio M.-A., Barco H., Schneider F. (2020). Direct Measurement of Mass and Economic Harvest and Post-Harvest Losses in Spanish Persimmon Primary Production. Agriculture.

[17] Page M.J., McKenzie J.E., Bossuyt P.M., Boutron I., Hoffmann T.C., Mulrow C.D., Shamseer L., Tetzlaff J.M., Akl E.A., Brennan S.E. (2021). The PRISMA 2020 statement: An updated guideline for reporting systematic reviews. BMJ.

[18] Xie C., Xie Z., Xu X., Yang D. (2015). Persimmon (*Diospyros kaki* L.) leaves: A review on traditional uses, phytochemistry and pharmacological properties. J. Ethnopharmacol..

[19] Kwon J., Park J.-E., Lee J.-S., Lee J.-H., Hwang H., Jung S.-H., Kwon H.-C., Jang D.-S. (2021). Chemical Constituents of the Leaves of *Diospyros kaki* (Persimmon). Plants.

[20] Lee J.-H., Lee Y.-B., Seo W.-D., Kang S.-T., Lim J.-W., Cho K.-M. (2012). Comparative Studies of Antioxidant Activities and Nutritional Constituents of Persimmon Juice (*Diospyros kaki* L. cv. Gapjubaekmok). Prev. Nutr. Food Sci..

[21] Sadiq Butt M., Tauseef Sultan M., Aziz M., Naz A., Ahmed W., Kumar N., Imran M., Muzaffarabad K. (2015). Persimmon (*Diospyros kaki*) fruit: Hidden phytochemicals and health claims. EXCLI J..

[22] Yaqub S., Farooq U., Shafi A., Akram K., Murtaza M.A., Kausar T., Siddique F. (2016). Chemistry and Functionality of Bioactive Compounds Present in Persimmon. J. Chem..

[23] Kawase M., Motohashi N., Satoh K., Sakagami H., Nakashima H., Tani S., Shirataki Y., Kurihara T., Spengler G., Wolfard K. (2003). Biological activity of persimmon (*Diospyros kaki*) peel extracts. Phytother. Res..

[24] Gorinstein S., Zachwieja Z., Folta M., Barton H., Piotrowicz J., Zemser M., Weisz M., Trakhtenberg S., Màrtín-Belloso O. (2001). Comparative Contents of Dietary Fiber, Total Phenolics, and Minerals in Persimmons and Apples. J. Agric. Food Chem..

[25] Zhang Y., Zhu L., Zhao M., Jia Y., Li K., Li C. (2024). The effects of inulin on solubilizing and improving anti-obesity activity of high polymerization persimmon tannin. Int. J. Biol. Macromol..

[26] Zillich O.V., Schweiggert-Weisz U., Eisner P., Kerscher M. (2015). Polyphenols as active ingredients for cosmetic products. Int. J. Cosmet. Sci..

[27] Sentandreu E., Cerdán-Calero M., Halket J.M., Navarro J.L. (2014). Rapid screening of low-molecular-weight phenols from persimmon (*Diospyros kaki*) pulp using liquid chromatography/UV–visible/electrospray mass spectrometry analysis. J. Sci. Food Agric..

[28] Rice-Evans C.A., Miller N.J., Paganga G. (1996). Structure-antioxidant activity relationships of flavonoids and phenolic acids. Free Radic. Biol. Med..

[29] Tsao R. (2010). Chemistry and Biochemistry of Dietary Polyphenols. Nutrients.

[30] Bei W., Peng W., Ma Y., Xu A. (2005). Flavonoids from the leaves of *Diospyros kaki* reduce hydrogen peroxide-induced injury of NG108-15 cells. Life Sci..

[31] Sun L., Zhang J., Lu X.-Y., Zhang L., Zhang Y. (2011). Evaluation to the antioxidant activity of total flavonoids extract from persimmon (*Diospyros kaki* L.) leaves. Food Chem. Toxicol..

[32] George A.P., Redpath S. (2008). Health and Medicinal Benefits of Persimmon Fruit: A Review. Adv. Hortic. Sci..

[33] Wang R., Shi X., Li K., Bunker A., Li C. (2023). Activity and potential mechanisms of action of persimmon tannins according to their structures: A review. Int. J. Biol. Macromol..

[34] Kripps K.A., Baker P.R., Thomas J.A., Skillman H.E., Bernstein L., Gaughan S., Burns C., Coughlin C.R., McCandless S.E., Larson A.A. (2021). REVIEW: Practical strategies to maintain anabolism by intravenous nutritional management in children with inborn metabolic diseases. Mol. Genet. Metab..

[35] Matsumoto K., Yokoyama S.-I., Gato N. (2009). Bile acid-binding activity of young persimmon (*Diospyros kaki*) fruit and its hypolipidemic effect in mice. Phytother. Res..

[36] Zou B., Ge Z.-Z., Zhang Y., Du J., Xu Z., Li C.-M. (2014). Persimmon Tannin accounts for hypolipidemic effects of persimmon through activating of AMPK and suppressing NF-κB activation and inflammatory responses in High-Fat Diet Rats. Food Funct..

[37] Zou B., Nie R., Zeng J.C., Ge Z.-Z., Xu Z., Dong H. (2014). Persimmon tannin alleviates hepatic steatosis in L02 cells by targeting miR-122 and miR-33b and its effects closely associated with the A type ECG dimer and EGCG dimer structural units. J. Funct. Foods.

[38] Zhu W., Lin K., Li K., Deng X., Li C. (2018). Reshaped fecal gut microbiota composition by the intake of high molecular weight persimmon tannin in normal and high-cholesterol diet-fed rats. Food Funct..

[39] Wilson I. (2021). Bile Acid-Induced Satiety to Treat Obesity. https://sites.dartmouth.edu/dujs/2021/06/07/bile-acid-induced-satiety-to-treat-obesity/.

[40] Yeung Y.T., Aziz F., Guerrero-Castilla A., Arguelles S. (2018). Signaling Pathways in Inflammation and Anti-inflammatory Therapies. Curr. Pharm. Des..

[41] Kitabatake M., Matsumura Y., Ouji-Sageshima N., Nishioka T., Hara A., Kayano S.-I., Ito T. (2021). Persimmon-derived tannin ameliorates the pathogenesis of ulcerative colitis in a murine model through inhibition of the inflammatory response and alteration of microbiota. Sci. Rep..

[42] Ding Y., Ren K., Dong H., Song F., Chen J., Guo Y., Liu Y., Tao W., Zhang Y. (2017). Flavonoids from persimmon (*Diospyros kaki* L.) leaves inhibit proliferation and induce apoptosis in PC-3 cells by activation of oxidative stress and mitochondrial apoptosis. Chem. Biol. Interact..

[43] Tapiero H., Townsend D.M., Tew K.D. (2004). The role of carotenoids in the prevention of human pathologies. Biomed. Pharmacother..

[44] Chen L.I., Guo Y., Alsaif G., Gao Y. (2020). Total Flavonoids Isolated from *Diospyros kaki* L. f. Leaves Induced Apoptosis and Oxidative Stress in Human Cancer Cells. Anticancer Res..

[45] Kashtoh H., Baek K.-H. (2022). Recent Updates on Phytoconstituent Alpha-Glucosidase Inhibitors: An Approach towards the Treatment of Type Two Diabetes. Plants.

[46] Sancheti S., Bafna M., Lee S.H., Seo S.Y. (2011). Persimmon leaf (*Diospyros kaki*), a potent α-glucosidase inhibitor and antioxidant: Alleviation of postprandial hyperglycemia in normal and diabetic rats. J. Med. Plants Res..

[47] Jung U.J., Park Y.B., Kim S.H., Choi M.-S. (2012). Supplementation of Persimmon Leaf Ameliorates Hyperglycemia, Dyslipidemia and Hepatic Fat Accumulation in Type 2 Diabetic Mice. PLoS ONE.

[48] Barthel A., Schmoll D. (2003). Novel concepts in insulin regulation of hepatic gluconeogenesis. Am. J. Physiol. Endocrinol. Metab..

[49] Sasaki K., Cripe T.P., Koch S.R., Andreone T.L., Petersen D.D., Beale E.G., Granner D.K. (1984). Multihormonal regulation of phosphoenolpyruvate carboxykinase gene transcription. The dominant role of insulin. J. Biol. Chem..

[50] Hussain A., Munir H., Saeed F. (2026). Ultrasound-Assisted Extraction of Banana and Water Chestnut Peel Phenolics for Improving the Storage Stability of Fish Patties. Food Innov. Funct. Sustain..

[51] Lee Y.A., Cho E.J., Tanaka T., Yokozawa T. (2007). Inhibitory Activities of Proanthocyanidins from Persimmon against Oxidative Stress and Digestive Enzymes Related to Diabetes. J. Nutr. Sci. Vitaminol..

[52] Silva V., Silva A., Ribeiro J., Aires A., Carvalho R., Amaral J.S., Barros L., Igrejas G., Poeta P. (2023). Screening of Chemical Composition, Antimicrobial and Antioxidant Activities in Pomegranate, Quince, and Persimmon Leaf, Peel, and Seed: Valorization of Autumn Fruits By-Products for a One Health Perspective. Antibiotics.

[53] Fu L., Lu W., Zhou X. (2016). Phenolic Compounds and In Vitro Antibacterial and Antioxidant Activities of Three Tropic Fruits: Persimmon, Guava, and Sweetsop. BioMed Res. Int..

[54] Amri Z., Zaouay F., Lazreg-Aref H., Soltana H., Mneri A., Mars M., Hammami M. (2017). Phytochemical content, Fatty acids composition and antioxidant potential of different pomegranate parts: Comparison between edible and non edible varieties grown in Tunisia. Int. J. Biol. Macromol..

[55] Arakawa H., Takasaki M., Tajima N., Fukamachi H., Igarashi T. (2014). Antibacterial Activities of Persimmon Extracts Relate with Their Hydrogen Peroxide Concentration. Biol. Pharm. Bull..

[56] Borges A., Ferreira C., Saavedra M.J., Simões M. (2013). Antibacterial Activity and Mode of Action of Ferulic and Gallic Acids Against Pathogenic Bacteria. Microb. Drug Resist..

[57] Jang I.-C., Oh W.-G., Ahn G.-H., Lee J.-H., Lee S.-C. (2011). Antioxidant activity of 4 cultivars of persimmon fruit. Food Sci. Biotechnol..

[58] Senter S.D., Chapman G.W., Forbus W.R., Payne J.A. (1991). Sugar and Nonvolatile Acid Composition of Persimmons During Maturation. J. Food Sci..

[59] Maeda H., Akagi T., Tao R. (2018). Quantitative characterization of fruit shape and its differentiation pattern in diverse persimmon (*Diospyros kaki*) cultivars. Sci. Hortic..

[60] Kaur N., Kumari A., Agarwal A., Sabharwal M., Dipti S. (2022). Utilisation of *Diospyros kaki* L. (persimmon) as a functional ingredient to produce functional foods: A review. Nutr. Food Sci..

[61] Lucas-González R., Ángel Pérez-Álvarez J., Moscaritolo S., Fernández-López J., Sacchetti G., Viuda-Martos M. (2021). Evaluation of polyphenol bioaccessibility and kinetic of starch digestion of spaghetti with persimmon (*Dyospyros kaki*) flours coproducts during in vitro gastrointestinal digestion. Food Chem..

[62] De Paula R., Rabalski I., Messia M.C., Abdel-Aal E.-S.M., Marconi E. (2017). Effect of processing on phenolic acids composition and radical scavenging capacity of barley pasta. Food Res. Int..

[63] Khan I., Yousif A., Johnson S.K., Gamlath S. (2013). Effect of sorghum flour addition on resistant starch content, phenolic profile and antioxidant capacity of durum wheat pasta. Food Res. Int..

[64] Rodriguez A., Cap M., Ramos N., Godoy F., Mascheroni R.H., Vaudagna S.R. (2019). High-pressure processing of persimmon purée: Stability during chilled storage. J. Food Process. Preserv..

[65] Ferrara L. (2021). Persimmon (*Diospyros kaki* L.): Nutritional importance and potential pharmacological activities of this ancient fruit. J. Softw. Eng. Simul..

[66] Hosseininejad S., González C.M., Hernando I., Moraga G. (2022). Valorization of Persimmon Fruit Through the Development of New Food Products. Front. Food Sci. Technol..

[67] de Ancos B., Gonzalez E., Cano M.P. (2000). Effect of High-Pressure Treatment on the Carotenoid Composition and the Radical Scavenging Activity of Persimmon Fruit Purees. J. Agric. Food Chem..

[68] Salazar-Bermeo J., Moreno-Chamba B., Heredia-Hortigüela R., Lizama V., Martínez-Madrid M., Saura D., Valero M., Neacsu M., Martí N. (2023). Green Technologies for Persimmon By-Products Revalorisation as Sustainable Sources of Dietary Fibre and Antioxidants for Functional Beverages Development. Antioxidants.

[69] Ben-Othman S., Jõudu I., Bhat R. (2020). Bioactives from Agri-Food Wastes: Present Insights and Future Challenges. Molecules.

[70] Devalaraja S., Jain S., Yadav H. (2011). Exotic fruits as therapeutic complements for diabetes, obesity and metabolic syndrome. Food Res. Int..

[71] Kayacan S., Karasu S., Akman P.K., Goktas H., Doymaz I., Sagdic O. (2020). Effect of different drying methods on total bioactive compounds, phenolic profile, in vitro bioaccessibility of phenolic and HMF formation of persimmon. LWT.

[72] Jia Y., Du J., Li K., Li C. (2022). Emulsification mechanism of persimmon pectin with promising emulsification capability and stability. Food Hydrocoll..

[73] Jia Y., Khalifa I., Dang M., Zhang Y., Zhu L., Zhao M., Li K., Li C. (2022). Confirmation and understanding the potential emulsifying characterization of persimmon pectin: From structural to diverse rheological aspects. Food Hydrocoll..

[74] Liu F., Zhou Z., Li G. (2020). Persimmon tannin functionalized polyacrylonitrile fiber for highly efficient and selective recovery of Au(III) from aqueous solution. Chemosphere.

[75] Dong Y., Yu X., Ye X., Gao Z., Wang S., Qu S. (2022). Current status and perspective of persimmon research in China. Technol. Hortic..

[76] Santos C.M., Abbade E.B., Fai A.E.C. (2023). Assessing land, nutrients and monetary value associated with postharvest loss of persimmon in Brazil: Pathways toward sustainability in agri-food systems. Br. Food J..

[77] Metibemu D.S., Ogungbe I.V. (2022). Carotenoids in Drug Discovery and Medicine: Pathways and Molecular Targets Implicated in Human Diseases. Molecules.

[78] Gato N., Kadowaki A., Hashimoto N., Yokoyama S.-I., Matsumoto K. (2013). Persimmon Fruit Tannin-Rich Fiber Reduces Cholesterol Levels in Humans. Ann. Nutr. Metab..

[79] Antony A., Farid M. (2022). Effect of Temperatures on Polyphenols during Extraction. Appl. Sci..

[80] Testoni A., Bellini E., Giordani E. (2002). Post-Harvest and Processing of Persimmon Fruit.

[81] Gu H.-F., Li C.-M., Xu Y.-J., Hu W.-F., Chen M.-H., Wan Q.-H. (2008). Structural features and antioxidant activity of tannin from persimmon pulp. Food Res. Int..

[82] Lim Y.J., Eom S.H. (2022). The Different Contributors to Antioxidant Activity in Thermally Dried Flesh and Peel of Astringent Persimmon Fruit. Antioxidants.

[83] Soares S., Brandão E., Guerreiro C., Soares S., Mateus N., de Freitas V. (2020). Tannins in Food: Insights into the Molecular Perception of Astringency and Bitter Taste. Molecules.

[84] Prinz J.F., Lucas P.W. (2000). Saliva tannin interactions. J. Oral Rehabil..

[85] Jain S., Ranganna G., Mohapatra S., Rathod M., Homeshvari N., Kore D.S., Saxena S., Anushi N. (2023). A Comprehensive Review on Persimmon (*Diospyros kaki*): Botanical, Horticultural, and Varietal Perspectives. Int. J. Environ. Clim. Change.

[86] Liu M., Wang J., Yang K., Qi Y., Zhang J., Fan M., Wei X. (2018). Optimization of ultrasonic-assisted extraction of antioxidant tannin from young astringent persimmon (*Diospyros kaki* L.) using response surface methodology. J. Food Process. Preserv..

[87] Uekert T., Singh A., DesVeaux J.S., Ghosh T., Bhatt A., Yadav G., Afzal S., Walzberg J., Knauer K.M., Nicholson S.R. (2023). Technical, Economic, and Environmental Comparison of Closed-Loop Recycling Technologies for Common Plastics. ACS Sustain. Chem. Eng..

[88] Asmy A.E., Rauf A., Rahmawaty, Badaruddin, Khasanah U. (2024). Reformulation of Persimmon Value-Added Model: Product Downstream Development Strategy for Farmers in East Java, Indonesia. Resources.

[89] Asmy A.E., Rauf A., Rahmawaty, Badaruddin, Khasanah U. (2024). Persimmon fruit downstream prospects: A promising solution to increase farmer income in Malang Regency, East Java, Indonesia. IOP Conf. Ser. Earth Environ. Sci..

[90] Katsieris D., Bhatnagar A., Yadav S., Achanta V., Harmes-Liedtke U., Rab S. (2024). Accreditation, Certification, and Inspection: Impact on Global Trade.

[91] Lezotre P.-L. (2023). International Cooperation, Convergence and Harmonization of Pharmaceutical Regulations.

