# Supplementary material for: Persimmon By-Products: Bioactive Phytochemicals, Sustainable Valorization and Potential Food and Health Applications
Source: Nutrients. 2026 Sep 27;18(19):3194. doi: 10.3390/nu18193194 (PMC13667039; doi:10.3390/nu18193194)
Supplement: Supplementary file 1 [file nutrients-18-03194-s001.zip › nutrients-4474046-supplementary.pdf]

PRISMA 2020 Checklist

Manuscript: Persimmon By-products: Bioactive Phytochemicals, Sustainable Valorization and Potential Food and Health Applications

Completed against the revised manuscript supplied to Nutrients. Items not reported or not applicable are identified explicitly rather than inferred.

Section and topic	Item #	PRISMA 2020 checklist item	Location where item is reported / status
TITLE<br>Title	1	Identify the report as a systematic review.	Not explicitly reported in the title. The manuscript is identified as a Review; the PRISMA 2020 reporting approach is stated in Section 2, Methodology (p. 5).
ABSTRACT<br>Abstract	2	See the PRISMA 2020 for Abstracts checklist.	p. 1, Abstract (partial reporting: topic, information sources, date range, principal findings, limitations and conclusions are reported; not all PRISMA for Abstracts elements are included).
INTRODUCTION<br>Rationale	3	Describe the rationale for the review in the context of existing knowledge.	pp. 3–5, Section 1, Introduction.
INTRODUCTION<br>Objectives	4	Provide an explicit statement of the objective(s) or question(s) the review addresses.	p. 5, final paragraph of Section 1, Introduction.
METHODS<br>Eligibility criteria	5	Specify the inclusion and exclusion criteria for the review and how studies were grouped for the syntheses.	pp. 5–6, Sections 2.1 (Inclusion Criteria) and 2.2 (Exclusion Criteria); grouping/synthesis described in Section 2.3.
METHODS<br>Information sources	6	Specify all databases, registers, websites, organisations, reference lists and other sources searched or consulted to identify studies. Specify the date when each source was last searched or consulted.	p. 5, Section 2, Methodology: Scopus, Web of Science, PubMed and ScienceDirect; publications from 2000 to March 2025; reference lists of key publications were also screened.
METHODS<br>Search strategy	7	Present the full search strategies for all databases, registers and websites, including any filters and limits used.	p. 5, Section 2, Methodology (partial). Search concepts/keywords and date range are reported, but complete database-specific reproducible search strings are not provided.
METHODS<br>Selection process	8	Specify the methods used to decide whether a study met the inclusion criteria of the review, including how many reviewers screened each record and each report retrieved, whether they worked independently, and, if applicable, details of automation tools used.	p. 6, Section 2.3, Study Screening and Data Extraction. Three authors screened records/full texts and reached decisions by consensus. Independent/duplicate screening and automation tools are not reported.
METHODS<br>Data collection process	9	Specify the methods used to collect data from reports, including how many reviewers collected data from each report, whether they worked independently, processes for obtaining or confirming data from study investigators, and, if applicable, details of automation tools used.	p. 6, Section 2.3. Three authors conducted screening/data extraction and the extracted information is described. Independent duplicate extraction, author contact and automation tools are not reported.
METHODS<br>Data items	10a	List and define all outcomes for which data were sought. Specify whether all results compatible with each outcome domain in each study were sought, and, if not, the methods used to decide which results to collect.	p. 6, Section 2.3 (partial). Biological activities and reported applications were extracted; formal outcome domains and rules for selecting among multiple results are not explicitly defined.
METHODS<br>Data items	10b	List and define all other variables for which data were sought. Describe any assumptions made about any missing or unclear information.	p. 6, Section 2.3: persimmon by-product/plant part, phytochemical composition, analytical and extraction methods, biological activities and reported applications. Assumptions for missing/unclear information are not reported.

Section and topic	Item #	PRISMA 2020 checklist item	Location where item is reported / status
METHODS<br>Study risk of bias assessment	11	Specify the methods used to assess risk of bias in the included studies, including details of the tool(s) used, how many reviewers assessed each study and whether they worked independently, and, if applicable, details of automation tools used.	Not reported. A formal risk-of-bias assessment was not described in the manuscript.
METHODS<br>Effect measures	12	Specify for each outcome the effect measure(s) used in the synthesis or presentation of results.	Not applicable to the narrative synthesis; no pooled effect estimates or meta-analysis were undertaken.
METHODS<br>Synthesis methods	13a	Describe the processes used to decide which studies were eligible for each synthesis.	pp. 5–6, Sections 2.1–2.3. Eligible studies were selected using predefined inclusion/exclusion criteria and organized by persimmon by-product type and functional properties.
METHODS<br>Synthesis methods	13b	Describe any methods required to prepare the data for presentation or synthesis, such as handling of missing summary statistics or data conversions.	Not reported / not applicable. No quantitative meta-analysis or data conversion procedure was described.
METHODS<br>Synthesis methods	13c	Describe any methods used to tabulate or visually display results of individual studies and syntheses.	Main text Sections 3–6; Table 2 (p. 12 onward), Table 3 (p. 23), Figures 2–4 and narrative presentation.
METHODS<br>Synthesis methods	13d	Describe any methods used to synthesize results and provide a rationale for the choice(s). If meta-analysis was performed, describe the model(s), methods to identify statistical heterogeneity, and software package(s) used.	p. 6, Section 2.3. Findings were synthesized qualitatively/narratively because study objectives, methodologies, compounds and outcomes were heterogeneous; quantitative meta-analysis was considered inappropriate.
METHODS<br>Synthesis methods	13e	Describe any methods used to explore possible causes of heterogeneity among study results.	No formal statistical investigation of heterogeneity was performed. Sources of variability are discussed qualitatively, particularly cultivar, tissue, extraction and methodological differences (Section 5; Section 7.2).
METHODS<br>Synthesis methods	13f	Describe any sensitivity analyses conducted to assess robustness of the synthesized results.	Not applicable; no quantitative synthesis or sensitivity analysis was performed.
METHODS<br>Reporting bias assessment	14	Describe any methods used to assess risk of bias due to missing results in a synthesis (arising from reporting biases).	Not reported; no formal reporting-bias assessment was performed.
METHODS<br>Certainty assessment	15	Describe any methods used to assess certainty (or confidence) in the body of evidence for an outcome.	Not reported; no formal certainty-of-evidence framework (e.g., GRADE) was applied.
RESULTS<br>Study selection	16a	Describe the results of the search and selection process, from the number of records identified in the search to the number of studies included in the review, ideally using a flow diagram.	p. 5, Section 2: 512 database records + 15 additional records; 251 duplicates removed; 276 screened; 89 full texts assessed; 53 full texts excluded; 36 studies included. Figure 1 (p. 7) presents the PRISMA 2020 flow diagram.
RESULTS<br>Study selection	16b	Cite studies that might appear to meet the inclusion criteria, but which were excluded, and explain why they were excluded.	Figure 1 (p. 7) reports grouped reasons for full-text exclusion; individual excluded studies that might appear eligible are not cited.
RESULTS<br>Study characteristics	17	Cite each included study and present its characteristics.	Sections 3–6 and Tables 2–3 present and cite characteristics/findings of the included literature (e.g., tissue, compounds, extraction/method, experimental model, biological activities and applications). A single table listing all 36 studies individually is not provided.

Section and topic	Item #	PRISMA 2020 checklist item	Location where item is reported / status
RESULTS<br>Risk of bias in studies	18	Present assessments of risk of bias for each included study.	Not reported; a formal risk-of-bias assessment was not performed.
RESULTS<br>Results of individual studies	19	For all outcomes, present, for each study: (a) summary statistics for each group (where appropriate) and (b) an effect estimate and its precision (e.g., confidence/credible interval), ideally using structured tables or plots.	Sections 3–6 and Tables 2–3 summarize individual study findings where available. Effect estimates with precision are not systematically reported because the review includes heterogeneous compositional, in vitro, in vivo and applied studies and no meta-analysis was undertaken.
RESULTS<br>Results of syntheses	20a	For each synthesis, briefly summarise the characteristics and risk of bias among contributing studies.	Characteristics are summarized narratively and in Tables 2–3; risk-of-bias assessments were not undertaken.
RESULTS<br>Results of syntheses	20b	Present results of all statistical syntheses conducted. If meta-analysis was done, present for each the summary estimate and its precision and measures of statistical heterogeneity. If comparing groups, describe direction of effect.	Not applicable; no statistical meta-analysis was conducted.
RESULTS<br>Results of syntheses	20c	Present results of all investigations of possible causes of heterogeneity among study results.	No formal statistical investigation was conducted. Qualitative differences related to cultivar, tissue, environmental conditions, extraction and assay methods are discussed in Section 5 and Section 7.2.
RESULTS<br>Results of syntheses	20d	Present results of all sensitivity analyses conducted to assess robustness of the synthesized results.	Not applicable; no sensitivity analyses were conducted.
RESULTS<br>Reporting biases	21	Present assessments of risk of bias due to missing results (arising from reporting biases) for each synthesis assessed.	Not reported; no formal reporting-bias assessment was performed.
RESULTS<br>Certainty of evidence	22	Present assessments of certainty (or confidence) in the body of evidence for each outcome assessed.	Not reported; no formal certainty-of-evidence assessment was performed.
DISCUSSION<br>Discussion	23a	Provide a general interpretation of the results in the context of other evidence.	Sections 3–7 and Section 9 (Conclusions, p. 27) provide interpretation of phytochemical composition, biological activities, applications and evidence limitations.
DISCUSSION<br>Discussion	23b	Discuss any limitations of the evidence included in the review.	Section 7.2, particularly Application Constraints (p. 25 onward), and Section 9 (p. 27): methodological variability, extraction differences, limited human clinical evidence, bioavailability and reproducibility limitations.
DISCUSSION<br>Discussion	23c	Discuss any limitations of the review processes used.	Not explicitly reported as limitations of the review process itself.
DISCUSSION<br>Discussion	23d	Discuss implications of the results for practice, policy, and future research.	Section 8, Future Directions (p. 26 onward), and Section 9, Conclusions (p. 27).
OTHER INFORMATION<br>Registration and protocol	24a	Provide registration information for the review, including register name and registration number, or state that the review was not registered.	Not reported in the manuscript.
OTHER INFORMATION<br>Registration and protocol	24b	Indicate where the review protocol can be accessed, or state that a protocol was not prepared.	Not reported in the manuscript.

Section and topic	Item #	PRISMA 2020 checklist item	Location where item is reported / status
OTHER INFORMATION Registration and protocol	24c	Describe and explain any amendments to information provided at registration or in the protocol.	Not applicable if the review was not registered and no protocol was prepared; this is not stated in the manuscript.
OTHER INFORMATION Support	25	Describe sources of financial or non-financial support for the review, and the role of the funders or sponsors in the review.	p. 29, Funding and Acknowledgments. Funding sources are reported (ARC DECRA DE220100055; University of Melbourne Collaborative Research Development Grant UoM-21/23); the role of funders/sponsors is not explicitly stated.
OTHER INFORMATION Competing interests	26	Declare any competing interests of review authors.	p. 29, Declaration of Competing Interest: authors declare no known competing financial interests or personal relationships.
OTHER INFORMATION Availability of data, code and other materials	27	Report which of the following are publicly available and where they can be found: template data collection forms; data extracted from included studies; data used for analyses; analytic code; any other materials used in the review.	Not reported. No public repository or data/materials availability statement for the review materials is provided.

Source and attribution: PRISMA 2020 checklist from Page MJ, McKenzie JE, Bossuyt PM, Boutron I, Hoffmann TC, Mulrow CD, et al. The PRISMA 2020 statement: an updated guideline for reporting systematic reviews. BMJ. 2021;372:n71. doi:10.1136/bmj.n71. Checklist distributed under CC BY 4.0.

Important note for submission: This checklist reflects only what is currently reported in the supplied manuscript. Entries marked 'Not reported' or 'partial' have not been filled by assumption.
